# Phospho-specific flow cytometry identifies aberrant signaling in indolent B-cell lymphoma

**DOI:** 10.1186/1471-2407-12-478

**Published:** 2012-10-16

**Authors:** Egil S Blix, Jonathan M Irish, Anne Husebekk, Jan Delabie, Lise Forfang, Anne M Tierens, June H Myklebust, Arne Kolstad

**Affiliations:** 1 Department of Oncology, University Hospital of North Norway, Tromsø, Norway; 2 Immunology Research group, Institute of Medical Biology, University of Tromsø, Tromsø, Norway; 3 Department of Medicine, Oncology Division, Stanford University, Stanford, CA 94305, USA; 4 Department of Microbiology and Immunology, Baxter Laboratory of Genetic Pharmacology, Stanford University, Stanford, CA 94305, USA; 5 Department of Laboratory Medicine, University Hospital North Norway, Tromsø, Norway; 6 Department of Pathology, Division of Cancer Medicine and Surgery, Oslo University Hospital, Oslo, Norway; 7 Institute for Cancer Research, Oslo University Hospital and Centre for Cancer Biomedicine, Oslo, Norway; 8 Department of Oncology, Norwegian Radium Hospital, Oslo University Hospital, Oslo, Norway

**Keywords:** B-cell lymphoma, B-cell receptor, CD40, Phospho-flow cytometry, Cell signaling

## Abstract

**Background:**

Knowledge about signaling pathways in malignant cells may provide prognostic and diagnostic information in addition to identify potential molecular targets for therapy. B-cell receptor (BCR) and co-receptor CD40 signaling is essential for normal B cells, and there is increasing evidence that signaling via BCR and CD40 plays an important role in the pathogenesis of B-cell lymphoma. The aim of this study was to investigate basal and induced signaling in lymphoma B cells and infiltrating T cells in single-cell suspensions of biopsies from small cell lymphocytic lymphoma/chronic lymphocytic leukemia (SLL/CLL) and marginal zone lymphoma (MZL) patients.

**Methods:**

Samples from untreated SLL/CLL and MZL patients were examined for basal and activation induced signaling by phospho-specific flow cytometry. A panel of 9 stimulation conditions targeting B and T cells, including crosslinking of the B cell receptor (BCR), CD40 ligand and interleukins in combination with 12 matching phospho-protein readouts was used to study signaling.

**Results:**

Malignant B cells from SLL/CLL patients had higher basal levels of phosphorylated (p)-SFKs, p-PLCγ, p-ERK, p-p38, p-p65 (NF-κB), p-STAT5 and p-STAT6, compared to healthy donor B cells. In contrast, anti-BCR induced signaling was highly impaired in SLL/CLL and MZL B cells as determined by low p-SFK, p-SYK and p-PLCγ levels. Impaired anti-BCR-induced p-PLCγ was associated with reduced surface expression of IgM and CD79b. Similarly, CD40L-induced p-ERK and p-p38 were also significantly reduced in lymphoma B cells, whereas p-p65 (NF-κB) was equal to that of normal B cells. In contrast, IL-2, IL-7 and IL-15 induced p-STAT5 in tumor-infiltrating T cells were not different from normal T cells.

**Conclusions:**

BCR signaling and CD40L-induced p-p38 was suppressed in malignant B cells from SLL/CLL and MZL patients. Single-cell phospho-specific flow cytometry for detection of basal as well as activation-induced phosphorylation of signaling proteins in distinct cell populations can be used to identify aberrant signaling pathways.

## Background

Small lymphocytic lymphoma/chronic lymphocytic leukemia (SLL/CLL) and marginal zone lymphoma (MZL) are indolent lymphoid malignancies that arise from mature B cells. The exact cellular origin of SLL/CLL is still controversial, although there is evidence that the lymphoma arises from memory B cells [1]. By contrast, splenic MZL possibly arises from naïve B cells whereas nodal MZL cells are presumed to develop from normal marginal zone B cells [2]. Therefore, signaling pathways that are required for normal B cell maturation and function are likely disturbed in SLL/CLL and MZL. These signaling pathways include the signals propagated through the B-cell receptor (BCR), CD40 and cytokine receptors.

BCR is important for the proliferation, differentiation and apoptosis of B cells [3]. Antigen stimulation via BCR in normal B cells initiates phosphorylation of the immunoreceptor tyrosine-based activation motifs (ITAMs) in the cytoplasmic tails of CD79a and CD79b. Phosphorylation of ITAMs is mediated by different Src family kinases (SFKs) including FYN, BLK, HCK, FGR, LCK and LYN [4]. The phosphorylated ITAMs serve as docking sites for SYK which is then phosphorylated at conserved tyrosine residues by SFKs. This activation initiates the coordinate assembly of the signalosome, composed of a variety of intracellular signaling molecules and includes BTK, phosphatidylinositol 3-kinase (PI3K), Vav and PLCγ. PLCγ activates PKC via DAG, and this further phosphorylates downstream signaling proteins like ERK, p38 and ultimately leads to activation of the pleiotropic transcription factor NF-κB (p65) [5]. The balance of these signals determines the B-cell fate [3]. There is now increasing evidence that signaling via BCR plays an important role in the pathogenesis of CLL [1]. However, there are conflicting results whether exposure to anti-IgM *in vitro* promotes or suppresses apoptosis in CLL cells [6] and other signals provided by the tumor microenvironment likely determines the outcome [7].

Activation of CD40, expressed by normal as well as malignant B cells, is an important co-stimulatory signal that enhances cell viability and promotes isotype class switching [8]. Furthermore, activation of CD40 on B cells induces expression of the co-stimulatory molecule B7 (CD80), improves presentation of alloantigen [9], and has been shown to activate NF-κB [10,11]. CD40-induced signaling in CLL cells results in up-regulation of NF-κB and activation of anti-apoptotic pathways [12-14], and induces drug resistance [15]. CD40 stimulation can also activate p38 in B-cell lymphoma cell lines [16]. Cytokine signaling in indolent B-cell lymphoma might also be important for lymphomagenesis, since IL-2 stimulation of CLL cells down-regulates p27 and forces the cells to traverse cell cycle [17]. However, conflicting results have been reported regarding IL-2 production in T cells from CLL patients [18,19].

Apart from providing information on potential molecular targets for therapy, the study of signaling pathways may provide prognostic and diagnostic information. Different properties of BCR signaling have been identified in normal B cells and in lymphoma B cells from follicular lymphoma patients [20,21], and impaired BCR signaling identified a subset of follicular lymphoma tumor cells with negative prognostic impact on patients overall survival [22]. Therefore, measuring phospho-proteins by flow cytometry to study signaling networks in cancer cells as well as in infiltrating immune cells at the single cell level [23,24] is feasible and relatively easy to introduce into clinical practice.

The aim of this study was to use phospho-specific flow cytometry to investigate basal and induced signaling in lymphoma B cells and infiltrating T cells in single-cell suspensions of biopsies from SLL/CLL and MZL patients. The results were compared with those of peripheral blood B cells and T cells from healthy donors (PBMC). We used 9 different stimuli targeting B- and T cells including CD40 ligand (CD40L), BCR engagement by F(ab’)_2_ (anti-BCR), interleukin 2 (IL-2), IL-7, and IL-15. The phospho-proteins studied included SFKs SYK, PLCγ, AKT, S6, ERK, p38, STAT1, STAT3, STAT5, STAT6 and NF-κB p65. Together, this yielded a comprehensive view of signaling networks in SLL/CLL and MZL lymphoma B cells and in tumor-infiltrating T cells.

## Methods

### Patients and healthy donors

The study was approved by Regional Committee for Medical Research Ethics (REK 2.2007.2949). Tumor biopsies from previously untreated patients with SLL/CLL (n = 11) and MZL (n = 5) were collected for diagnostic purposes. Left-over samples were used for this study after informed consent from the patients. Single-cell suspensions were prepared from tumor biopsies and stored in liquid nitrogen in cryotubes until used. All biopsies were reviewed and subtyped according to the WHO classification [25] by a hematopathologist.in 2010. Peripheral blood was drawn from consenting healthy blood donors (n=9) and PBMC were isolated by density gradient centrifugation (Lymphoprep Axis-Shield, Dundee, United Kingdom). Cells were stored in liquid nitrogen in cryotubes until used.

### Reagents and Antibodies

Anti-BCR antibodies were a mixture of goat polyclonal anti-human IgM F(ab’)_2_ and goat polyclonal anti-human IgG F(ab’)_2_ (Invitrogen Carlsbad, CA, USA); each at a final concentration of 10 μg/ml. H_2_O_2_ (Sigma-Aldrich, Oslo, Norway) was used at a final concentration of 3.3 mM. The cells were stimulated with IL-2 at 500 U/ml, IL-7 at 20 ng/ml, IL-15 at 20 ng/ml, and soluble CD40 ligand at 200 ng/ml (all from Peprotech, Rocky Hill, NJ, USA). Phorbol 12-Myristate 13-Acetate (PMA) and ionomycin (both from Sigma-Aldrich, Saint Louis, Missouri, USA) were used at 1 μg/ml each.

Anti-CD20-PerCPCy5.5 (clone SK7), anti-CD5-PE-Cy7 (clone L17F12), anti-p-PLCγ2-Alexa488 (Y759), anti-p-ERK1/2-Alexa488 (T202/Y204), anti-p-SYK / Zap70-Alexa647 (Y352/Y319), anti-p-STAT1-Alexa488 (Y701), anti-p-STAT6-Alexa647 (Y641), anti-p-p38-Alexa488 (T180/Y182), anti-p-STAT3-Alexa647 (Y705), anti-p-LCK-Alexa488 (Y505), anti-p-STAT5-Alexa647 (Y694) and anti-p-65(NF-κB)-Alexa488 (Ser529) were all from BD Biosciences (San Jose, CA, USA). The antibodies anti-p-S6-Alexa647 (Ser235/236) and anti-p-AKT-Alexa647 (Ser473) were from Cell Signaling Technology (Danvers, MA, USA). The following Abs were used for immune phenotypic analysis: CD3 PacBlue (UCHT1), CD5 PE-Cy7 (L17F12), CD20 APC-H7 (L27), CD79a APC (HM47), CD79b PE (SN8) and CD23 PerCPCY5.5 (M-L233) were from BD, and IgM FITC (AHI1608), IgG FITC (AHI1308), IgL PE (AHI1907) and IgK APC (MH10515) were from Invitrogen.

### Activation of Signaling and Fluorescent cell barcoding

Individual cryotubes were thawed, cells were washed in RPMI, counted and incubated for 30 minutes in a tissue culture incubator at 37°C with 5% CO_2_. After this initial incubation, 200 μl of the cell suspension was aliquoted into a 96-well plate, and the cells were incubated an additional 45 minutes at 37°C. Cells were then left unstimulated or activated with anti-BCR, H_2_O_2,_ IL-2, IL-7, IL-15, soluble CD40 ligand or with PMA and ionomycin for 4, 15 or 45 minutes.

Signaling was stopped by fixation in paraformaldehyde (Electron Microscopy Service, Hatfield, PA, USA) for 5 minutes at a final concentration of 1.5% at room temperature. The cells were pelleted by high speed centrifugation at 800 g, resuspended in PBS and permeabilized in 90% methanol at −20°C for at least 10 minutes. The cells where then pelleted by high speed centrifugation at 800 g and resuspended in PBS.

Fluorescent cell bar coding were then performed as previously described [26]. Briefly, cells in each well (=one stimulation condition) were stained with a unique combination of two different fluorescent esters; Pacific Blue and Pacific Orange (Invitrogen AS, Oslo, Norway), each used at 3 different concentration level. This bar coding made it possible to identify 3x3 different cell populations (i.e. all the different stimulation conditions given to one patient sample). Pacific Blue was used at a final concentrations of 0.000780, 0.00702 or 0.0498, ng/μL and Pacific Orange was used at 0.00870, 0.0870 or 0.522 ng/μL. Labeling was stopped by adding PBS w/ 1% BSA and then pelleted by high speed centrifugation (800 g), resuspended in PBS with 1% BSA (Sigma-Aldrich, Oslo, Norway) and combined in one tube per individual patient sample.

### Flow cytometry

The barcoded cells were aliquoted into six tubes for staining with different antibody panels. Each panel contained a backbone of the antibodies anti-CD20 and anti-CD5 in addition to two different phospho-antibodies. The cells were stained for 30 minutes in the dark at 4°C, pelleted by centrifugation at 350 g and resuspended in PBS.

For phenotypic analysis, freshly thawed patient samples were stained with various antibodies for 30 minutes in the dark at 4°C, pelleted by centrifugation at 350 g and resuspended in PBS. For staining with CD79a (intracellular epitope), the cells were fixed and permeabilized using paraformaldehyde and 90% methanol as described above, prior to Ab staining.

Acquisition was performed with a three-laser flow cytometer (FACSAria or LSR II, Becton Dickinson, Franklin Lakes, NJ, USA). Data were collected and analyzed using BD FACSDiva software and Cytobank Software ( http://www.Cytobank.org), respectively. Only data from samples of which at least 50% of cells responded to any of the stimulation condition were included.

### Statistical analysis

GraphPad Software (La Jolla, CA, USA) was used to determine statistical significance of difference between groups by applying statistical tests as specified in results.

### Cluster analysis

Hierarchical cluster analysis of flow cytometry data were performed by the open source programs Cluster [27] and Treeview [28] , with the use of complete linkage.

## Results

### Phospho-specific flow cytometry identifies different signaling characteristics within cell subsets in lymphoma biopsies

**Figure 1:**
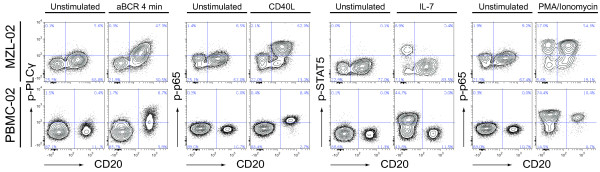
**Basal and activation induced signaling in samples from lymphoma patients and healthy donors.** Samples from lymphoma patients or from peripheral blood of healthy donors (PBMCs) were stimulated with various stimulation conditions (unstimulated, 4 minutes anti-(a)BCR (crosslinking with anti-IgM/anti-IgG), or 15 minutes CD40L, IL-7 or PMA/ionomycin), followed by measurement of phospho-proteins using flow cytometry**.** Contour plots show basal level (unstimulated) or activation-induced levels of p-PLCγ, p-p65 and p-STAT5, in a marginal lymphoma patient sample (MZL-02) and a healthy donor PBMC (PBMC-02).

To examine whether intracellular signaling was altered in samples from SLL/CLL and MZL patients, we combined surface markers and phospho-protein specific antibodies and detected basal as well as activation-induced signaling by flow cytometry. Malignant B-cells were identified by their expression of CD20 and CD5, and could be separated from CD20^-^CD5^+^ infiltrating T cells. Crosslinking of BCR with a mixture of anti-IgM and anti-IgG Abs (aBCR) induced p-PLCγ only in B cells (Figure 1). Similarly, CD40L induced p-p65 only in B cells (Figure 1). IL-7 induced p-STAT5 in T cells only, whereas PMA/ionomycin induced p-p65 in both cell types (Figure 1). Together, these results show that signaling responses can be studied in malignant B cells and distinguished from those of infiltrating T cells.

### Elevated basal levels of phospho-proteins in malignant B cells from SLL/CLL patients

**Figure 2:**
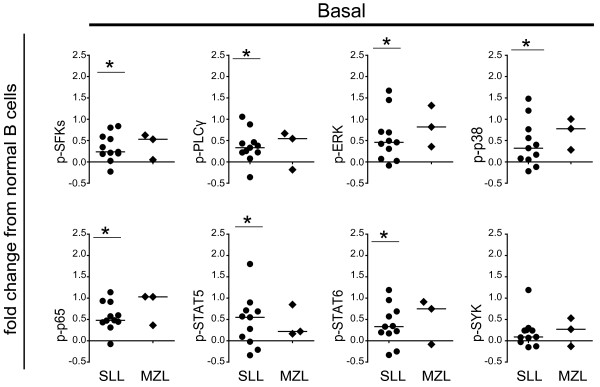
**Malignant B cells in SLL/CLL have higher basal levels of phospho-proteins.** Scatter plots of the basal level of 8 phospho-proteins in malignant B cells from SLL/CLL patients (SLL/CLL, n = 11), MZL patients (MZL, n = 3). Unstimulated samples were used for analysis of basal level of phospho-proteins. MFI for each phospho-protein in the CD20^+^CD5^+^ (SLL/CLL) or CD20^+^ subset (MZL) were normalized to MFI in the CD20^+^ B cell subset from healthy blood donor PBMC analyzed at the same time. * indicates significant change from normal CD 20^+^ B cells, p < 0.05; Wilcoxon Signed Rank test.

First, we asked whether there were differences in the basal phospho-protein levels in malignant B cells from SLL/CLL or MZL patients, relative to healthy donor B cells. For this purpose, we analyzed samples from 11 SLL/CLL patients and 3 MZL patients where flow cytometry data from healthy donor PBMCs, analyzed at the same time, were available. The fold change in median fluorescence intensity (MFI) for phospho-proteins in unstimulated malignant B cells from SLL/CLL or MZL patients were normalized to the MFI in CD20^+^ B cells from peripheral blood B cells from healthy donors (PBMC). We found significantly higher basal levels of the following phospho-proteins in malignant B cells from SLL/CLL patients: p-SFKs, p-PLCγ, p-ERK, p-p38 and p- p65 (NF-κB), p-STAT5 and p-STAT6 (Figure 2). Of note, the basal levels of phospho-proteins were heterogeneous in patients samples as many of them showed only small elevations (i.e. fold change <0.5), in contrast to a few which had high basal levels (Figure 2). We also observed higher levels of p-SYK in most SLL/CLL patients, but this finding did not reach statistical significance (p=0.068). Overall, we observed higher basal levels of several phospho-proteins in malignant B cells from SLL/CLL patients.

### Impaired, but sustained BCR signaling in SLL/CLL and MZL tumor B cells

**Figure 3:**
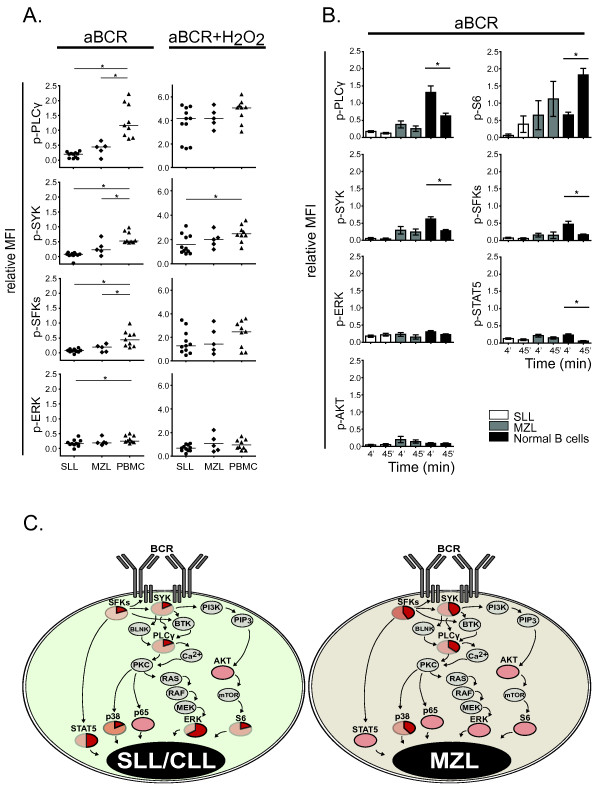
**BCR induced signaling is impaired in SLL/CLL and MZL lymphoma B cells.** Flow cytometry analysis of BCR-induced phosphorylation of key signaling proteins downstream of BCR. Lymphoma patient samples and PBMCs from healthy donors were stimulated with BCR crosslinking alone (anti-IgM/anti-IgG) or with a combination of BCR crosslinking and hydrogen peroxide (H_2_O_2_) for 4 or 45 minutes. (**A**) Scatter plot showing relative MFI for each aBCR- or aBCR+H_2_O_2_-induced phospho-proteins in CD20^+^ B cells after 4 minutes, normalized to the MFI value in the corresponding unstimulated CD20^+^ B cells from the same individual. SLL/CLL (n = 11), MZL (n = 5) and PBMC (n = 9); * p < 0.05; Mann Whitney test. (**B**) BCR induced phosphorylation after 4 and 45 minutes. Relative MFI was calculated as in A), and shown is bar charts of mean relative MFI ± SEM, for SLL/CLL (n = 11); MZL (n = 5) and PBMC (n = 9). * p < 0.05; paired t test. (**C**) Overview of phosphorylated signaling proteins after 4 minutes of BCR stimulation. Phospho-proteins investigated have red color. Pie charts indicate phospho-proteins with significant reduced expression in SLL/CLL or MZL compared to normal B cells. The dark red color in pie charts indicates percentage of relative MFI in SLL/CLL or MZL compared to normal B cells. Phospho-proteins with only light red color indicate no significant change of expression compared to normal B cells.

Activation of BCR is important for survival and proliferation of normal as well as malignant B cells. BCR activation of SLL/CLL cells can increase the level of the anti-apoptotic protein MCL-1 and subsequent resistance to fludarabine, or induce down-regulation of MCL-1 and induction of apoptosis, depending on the nature of BCR stimulation [29] . We therefore assessed phosphorylation of several signaling proteins downstream of BCR, 4 and 45 minutes post BCR cross-linking, relative to unstimulated B cells from the same individual. In normal B cells, of the phospho-proteins analyzed, p-PLCγ showed the largest increase after activation of BCR (Figure 3A). Strikingly, BCR-induced phosphorylation of p-PLCγ was significantly lower in the malignant B cells from SLL/CLL and MZL patients, compared to healthy donor B cells, with an 83% and 62% reduction in median MFI, respectively. Importantly, phosphorylation of SYK/Zap70 and SFKs after 4 minutes of BCR stimulation was also significantly impaired in SLL/CLL and MZL cells (Figure 3A). Phosphorylation of SYK/Zap70 was reduced by 85% and 56%, whereas phosphorylation of SFK was reduced by 82% and 57% in SLL/CLL and MZL, respectively. In comparison, there were only minor changes in p-ERK after BCR stimulation in the lymphoma cells, compared to normal B cells. Originally, CLL was thought to be derived from CD5^+^ B cells [1].We tested if CD5^+^CD20^+^ B cells from healthy donors also had suppressed anti-BCR-induced signaling, compared to CD5^-^CD20^+^ B cells. However, we found no difference in p-SFKs, p-SYK, p-PLCγ and p-ERK expression at 4, 15 or 45 minutes of anti-BCR activation (Additional file 1: Figure S1), suggesting that peripheral blood CD20^+^ B cells serves as a relevant normal counterpart.

BCR signaling is controlled by protein tyrosine phosphatases (PTPs) that dephosphorylate signaling molecules after activation in order to terminate signaling. Hydrogen peroxide (H_2_O_2_) regulates the quantity and length of signaling by inhibiting BCR-induced PTP activity [20]. When H_2_O_2_ was added immediately after BCR cross-linking, BCR-induced signaling was restored in lymphoma B cells, as BCR and H_2_O_2_-induced p-PLCγ, p-SFKs and p-ERK were no longer significantly different from healthy donor B cells (Figure 3A). Thus, these results suggest that lymphoma B cells have impaired BCR-induced signaling, but that inhibition of phosphatases can restore signaling in these cells. Furthermore, in normal B cells, all investigated phospho-proteins except p-S6, showed higher expression levels at 4 minutes compared to 45 minutes after BCR activation (Figure 3B). Delayed S6 phosphorylation with strongest activation 45 minutes after BCR cross-linking was also evident in SLL/CLL and MZL cells. Normal B cells had a significant decrease in levels of phosphorylated PLCγ, SYK, SFKs and STAT5 from 4 to 45 minutes, in contrast to SLL/CLL and MZL malignant B cells which showed no significant decrease (Figure 3B). Thus, malignant B cells from SLL/CLL and MZL had low, but sustained BCR-induced signaling. Based on the observed results, a model for BCR-induced signaling in lymphoma B cells from SLL/CLL and MZL patients was constructed (Figure 3C).

### Low CD79b expression correlates with impaired BCR-induced p-PLCγ

**Figure 4:**
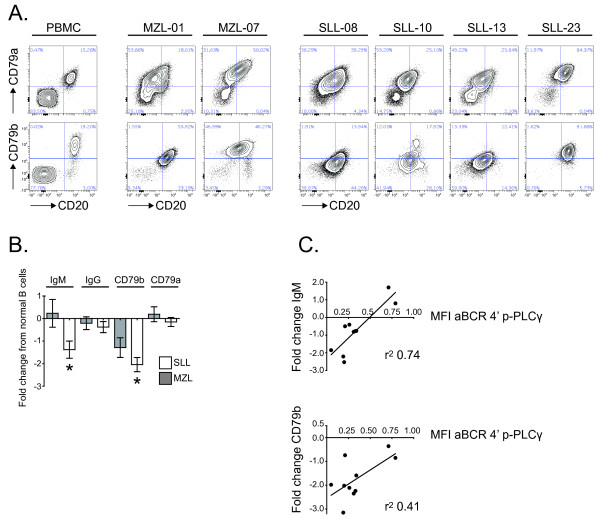
**Low CD79b and IgM expression in SLL/CLL cells correlates with impaired aBCR-induced p-PLCγ.** (**A**) Contour plot showing CD20 and CD79b expression in CD20^+^ B cells from representative normal PBMC, MZL (n=2) and SLL/CLL (n=4). (**B**) Bar chart illustrating expression of IgM, IgG, CD79b and CD79a as median fold change MFI ± SEM in SLL/CLL (n=6) and MZL (n=4) as fold change MFI from normal CD20^+^ B from PBMCs analyzed in parallel. * p < 0.05. (**C**) Scatter plot of p-PLCγ MFI after 4 minutes of BCR stimulation in SLL/CLL and MZL malignant CD20^+^ B cells on x axis and IgM or CD79b fold change MFI in SLL/CLL and MZL cells from normal CD20^+^ B cells on y axis.

We next investigated if impaired BCR signaling in malignant B cells from SLL/CLL or MZL patients could be explained by loss or reduced cell surface levels of IgM, CD79a or CD79b. A subgroup of SLL/CLL (n=6) and MZL (n=4) patients were analyzed for this purpose (Table 1). Expression of CD79b was frequently found to be down-regulated in SLL/CLL and MZL patients, in contrast to a more uniform expression of CD79a (Figure 4A). Indeed, surface expression of CD79b and IgM were greatly reduced in SLL/CLL (p=0.0013 and p=0.015, respectively), compared to healthy donor CD20^+^ B cells (Figure 4B). Importantly, in malignant B cells from SLL/CLL patients, BCR-induced p-PLCγ was correlated with CD79b or IgM expression levels (Figure 4C; r^2^=0.41 and r^2^=0.74, respectively). Altogether, these results suggest that impaired BCR-induced signaling in SLL/CLL lymphoma B cells is due to low surface expression of IgM and CD79b.

**Table 1:** Immunophenotyping data in SLL/CLL and MZL

**Lymphoma type**	**Mean MFI relative to normal B cells (SEM)**			
	**IgM**	**IgG**	**CD79a**	**CD79b**
**MZL**	0.23 (0.62)	−0.21 (0.28)	0.19 (0.33)	−1.29 (0.43)
**SLL/CLL**	−1.38 (0.38)	−0.38 (0.24)	−0.15 (0.18)	−2.04 (0.32)

Expression of immunoglobulin and CD79 subclasses in SLL/CLL and MZL compared to normal B cells presented as MFI fold change from normal B cells.

### Impaired CD40 signaling in SLL/CLL and MZL cells

**Figure 5:**
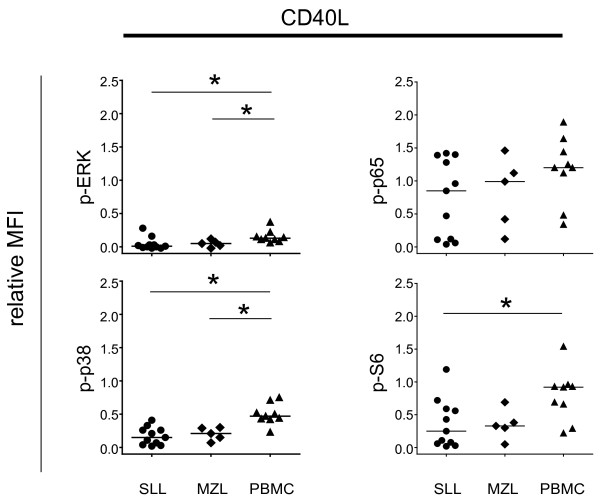
**Impaired phosphorylation of p38 and ERK after CD40L stimulation in SLL/CLL samples.** Lymphoma patient samples and PBMCs from healthy donors where stimulated with CD40L for 15 minutes and the expression of different phospho-proteins was measured by flow cytometry as indicated. Shown is a scatter plot of MFI in the CD20^+^CD5^+^/CD20^+^-subset, relative to the corresponding unstimulated sample from the same individual. SLL/CLL (n = 11), MZL (n = 5) and PBMC (n = 9); * p < 0.05; Mann Whitney test.

In order to investigate CD40-induced signaling in malignant B cells from SLL/CLL and MZL patients, phosphorylation of signaling proteins were assessed 15 minutes after CD40L stimulation. In healthy donor B cells, CD40L induced the strongest increase in p-p65, p-S6 and p-p38 (Figure 5). In contrast, malignant B cells from SLL/CLL and MZL had significantly less CD40L-induced p-p38 and p-ERK, compared to normal B cells. Mean relative MFI levels of p-p38 in SLL/CLL and MZL were 68% and 55% lower than in normal B cells, respectively. Malignant B-cells from SLL/CLL also had impaired CD40L-induced p-S6 (Figure 5). In contrast, no significant difference was observed in CD40L-induced p-p65. Altogether, CD40L signaling was clearly impaired in malignant B cells from SLL/CLL and MZL patients as a key feature was diminished p38 phosphorylation.

### Tumor-infiltrating T cells demonstrate similar interleukin-induced STAT5 signaling as normal T cells

**Figure 6:**
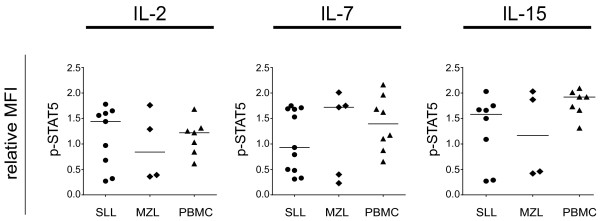
**Cytokine induced p-STAT5 are heterogeneous in tumor-infiltrating T cells.** The SLL/CLL, MZL and PBMC samples were stimulated with cytokines as indicated for 15 minutes. Scatter plot of MFI of p-STAT5 in stimulated relative to unstimulated CD5+CD20- T cells, SLL/CLL (n = 11), MZL (n = 5) and PBMC (n = 9); * p < 0.05; Mann Whitney test.

In order to assess whether signaling responses also were altered in tumor-infiltrating T cells in SLL/CLL and MZL, tumor samples were stimulated with IL-2, IL-7 and IL-15 for 15 minutes and phospho-protein levels were evaluated in the CD5^+^ CD20^-^ T cell fraction (Figure 6). In general, no significant differences in cytokine-induced p-STAT5 were observed in tumor-infiltrating T cells in SLL/CLL and MZL samples, compared to T cells from healthy donors. It should be noted that cytokine-induced signaling were markedly heterogeneous in tumor-infiltrating T cells in the SLL/CLL and MZL groups (Figure 6). Taken together, tumor-infiltrating T cells in SLL/CLL samples displayed similar cytokine-signaling responses as compared to healthy donor T cells.

### Unsupervised cluster analysis of phospho-protein expression profiles of SLL/CLL and MZL biopsies compared to normal peripheral blood

**Figure 7:**
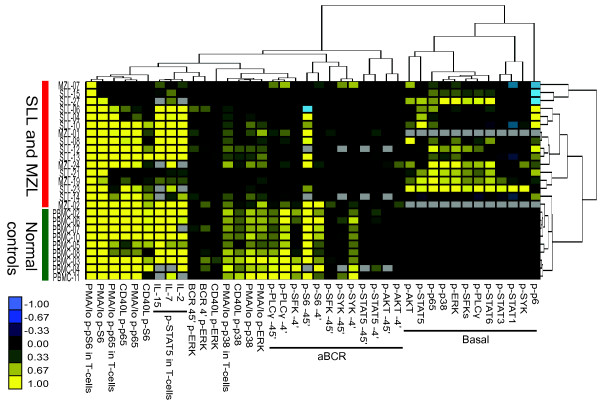
**Unsupervised cluster analysis of phospho-proteins depicts that lymphoma samples can be separated from normal PBMCs.** All phospho-proteins with a relative MFI greater or less than 0.25 in the basal or induced conditions were included in the unsupervised cluster analysis. Phospho-proteins and samples were subjected to clustering using complete linkage method**.** The heatmap illustrates basal and induced MFI phospho-proteins in all SLL/CLL, MZL patients and PBMCs. Basal, unstimulated MFI represent a ratio of MFI in the lymphoma CD20^+^-subset and MFI in CD20^+^ normal B cells. Induced signaling are the ratio of MFI in the stimulated CD20^+^-subset or the CD5^+^ CD20^-^-subset in case of T cells, and MFI from unstimulated cells in the same subset. Missing data are indicated by grey color.

We finally asked whether SLL/CLL and MZL could be separated from healthy donors using unsupervised cluster analysis, based on basal and activation-induced phospho-protein levels. Unsupervised cluster analysis revealed that samples from healthy donors clustered together, whereas samples from SLL/CLL and MZL made several different clusters (Figure 7). One of the clusters contained patient samples with higher basal levels of several phospho-proteins, but had low cytokine-induced p-STAT5 T-cell responses, whereas two other clusters had low basal levels of phospho-proteins, but high cytokine-induced p-STAT5 T cell responses. Whether the different clusters identified can be translated into meaningful clinical subclasses, will require similar analysis in a larger patient cohort.

## Discussion

In this study we used phospho-specific flow cytometry to map differences in signaling properties within the B- and T-cell subsets from SLL/CLL and MZL patient samples. We found increased basal levels of several phospho-proteins in lymphoma B cells, whereas they overall had impaired, but sustained anti-BCR-induced p-PLCγ, p-SYK/Zap70, p-SFKs and p-ERK, compared to healthy donor B cells. Importantly, impaired BCR-induced p-PLCγ was associated with reduced surface expression of IgM and CD79b. Additional signaling aberrations in lymphoma B cells included CD40L-induced p-p38 and p-ERK.

Overall, malignant B cells from SLL/CLL patients showed significant higher basal levels of several phospho-proteins, including p-SFKs, p-PLCγ, p-ERK, p-p38, p-p65 (NF-κB), p-STAT5 and p-STAT6, but the levels varied considerably between different patients. Constitutive active STATs in SLL/CLL and MZL potentially have biological significance, as targeting JAK/STAT pathways had therapeutic benefits in relapsed lymphomas [30]. JAK2 inhibition by SB1518 prevented tyrosine phosphorylation of STAT proteins, leading to cell cycle arrest and induction of apoptosis [31]. Higher levels of basal p-ERK and p-p38 in SLL/CLL lymphoma cells are also in agreement with previous reports [32,33]. Earlier work with primary CLL samples have shown constitutive phosphorylation of the SFK LYN, relative to normal B cells [34] and constitutive phosphorylation of SYK relative to cell lines [35,36]. We also observed higher basal levels of p-SFKs, whereas basal p-SYK levels did not reach statistical significance, possibly due to low number of patients. The biological significance of basal p-SYK in CLL has clinical relevance, since SLL/CLL patients treated with the SYK inhibitor R406 has shown promising response rates [37]. Whether the lymphoma patients whose lymphoma B cells’ have high basal levels of signaling proteins such as p-SFK and p-SYK, also are the ones with the greatest clinical responses upon specific kinase inhibitor therapy, should be the focus of future studies.

We found that BCR-induced p-SFK, p-SYK/p-Zap70, p-PLCγ and p-ERK were highly impaired in SLL/CLL and MZL lymphoma B cells, compared to normal B cells. Furthermore, we found reduced levels of surface IgM and CD79b in CLL/SLL lymphoma cells, and this correlated with impaired anti-BCR-induced p-PLCγ. Low expression of CD79b in CLL cells has also been reported previously [38]. Furthermore, CLL tumor cells which are unresponsive to anti-IgM, can respond to anti-CD79a treatment, indicating a deficit in signal transmission from the BCR to CD79a/b [36]. However, since a subgroup of CLL samples was unresponsive to activation with anti-CD79a, a potential defect further downstream in the BCR signaling pathway is also possible [36]. Surface IgM expression varies considerably among primary CLL samples, with a subset of patients having markedly decreased IgM expression on the malignant cells [39-41]. Anti-IgM stimulation in primary CLL samples results in global tyrosine phosphorylation mainly in unmutated CLL, but not in mutated CLL samples [36]. The differential response to BCR stimulation in unmutated vs. mutated CLL has been confirmed by other groups [42,43]. We did not find significant different BCR-induced phosphorylation of target proteins (SFKs, SYK, PLCγ, ERK and STAT5) between unmutated and mutated SLL/CLL, possibly due to small sample size (data not shown). Further, ZAP-70 expression can enhance BCR signaling after anti-IgM treatment, independent of its kinase activity [44], and CLL cells that expressed ZAP-70 had significantly higher levels of phosphorylated CD79b compared to CLL lacking ZAP-70.

CD40L-induced signaling was also impaired in SLL/CLL and MZL lymphoma B cells compared to normal B cells with significant lower phosphorylation of p38, ERK and S6 in SLL/CLL and lower p38 and ERK in MZL. This finding is in line with previous observations were CD40L stimulation resulted in diminished protein tyrosine kinase phosphorylation in CLL B cells compared to normal B cells, despite similar expression levels of CD40 [45]. The reason for diminished p-p38 expression in SLL/CLL and MZL lymphoma B cells is unclear. Activation of p38 has a pro-apoptotic function in CLL cells, and earlier work has shown that rituximab-induced apoptosis is dependent on phosphorylation of p38 [46]. Furthermore, recent work in primary CLL cells illustrates that chemotherapy-induced up-regulation of the pro-apoptotic protein NOXA is at least partly dependent on p38 [47]. Thus, loss of p38 function is likely to give the tumor cells a survival advantage. Therefore, the role of p-p38 in B-cell malignancies warrants further investigation. In contrast to the overall weak CD40L induced phosphorylation of p38 in malignant B cells, p-p65 (NF-κB) expression was markedly heterogeneous in SLL/CLL and MZL. This finding is in accordance with earlier work where lymphoma B cells from CLL patients were heterogeneous in basal- as well as activation-induced NF-κB [48]. This has potentially clinical implications as a correlation between the NF-κB subunit Rel-A (p65) DNA binding in CLL cells and lymphocyte doubling time was identified, and Rel-A DNA binding was positively correlated with in vitro resistance to fludarabine [48].

CD40 stimulation resulted in strong phosphorylation of S6 in MZL cells, in a subset of SLL/CLL samples, and in normal B cells. Interestingly, induction of p-S6 seemed to be partially independent of PI3K since we did not find an increased p-AKT level after stimulation with CD40L. Similarly, in SLL/CLL B-lymphoma cells BCR activation resulted in high levels of p-S6, with only small increase in p-AKT. PI3K-independent activation of mTor has previously been described in transformed B cells [49]. Furthermore, it was previously shown that marginal zone B cells expressed more PI3K independent p-S6 after BCR stimulation than follicular B cells [50]. Inhibition of mTor is a potential target in cancer therapy, and clinical trials with its inhibitor everolimus have shown promising results in CLL [51]. Thus, the marked, but variable levels of p-S6 in both unstimulated (data not shown) and CD40L activated cells suggests that detection of p-S6 could be tested as a biomarker in clinical trials with mTor inhibitors.

Tumor-infiltrating T cells in SLL/CLL and MZL showed no significant differences in cytokine induced STAT5 phosphorylation, compared to normal T cells. This was in contrast to ligand independent stimulation which resulted in decreased phosphorylation of p38 and NF-κB p65 in the former. Earlier work with tumor-infiltrating T cells from CLL patients demonstrates defective immunological synapse formation with antigen presenting cells together with reduced tyrosine-phosphorylated proteins at the synapse [52]. Of interest, we found a marked variability in cytokine-induced signaling between SLL/CLL and MZL cell samples. Whether this has prognostic significance for these diseases, remains to be demonstrated.

## Conclusions

In conclusion, this work has provided a better overview of basal, BCR- and CD40L-induced signaling in lymphoma B cells as well as cytokine-induced signaling responses in infiltrating T cells in patients with SLL/CLL and MZL. Although we did not identify unique signaling profiles that could distinguish SLL/CLL from MZL, we identified contrasting signaling abnormalities in the lymphoma B cells compared to normal B cells. Further studies using single-cell phospho-specific flow cytometry to obtain patient specific signaling aberrations could provide an opportunity to personalize inhibitor treatment in B cell lymphoma patients.

## Competing interests

The authors declare that they have no competing interests.

## Authors’ contributions

ESB, JMI, JHM, AH and AK designed study; ESB, LF and AT conducted experiments; ESB, JMI and JHM analyzed data, JD validated all patients’ diagnosis; A.K provided clinical data; ESB drafted the manuscript and all authors participated in discussion of results and approved final manuscript.

## Pre-publication history

The pre-publication history for this paper can be accessed here:

http://www.biomedcentral.com/1471-2407/12/478/prepub

## Supplementary Material

Additional file 1 **Figure S1. No difference in anti-BCR induced signaling between CD20**^**+**^**CD5**^**-**^**and CD20**^**+**^**CD5**^**+**^**B cells.** PBMCs (n=4) from healthy donors were stimulated with aBCR for 4, 15 or 45 minutes. Flow cytometry analysis of p-SFKs, p-SYK, p- PLCγ and p-S6 after gating on CD20^+^CD5^+^ or CD20^+^CD5^-^ cells. (A) MFI in aBCR stimulated cells relative to unstimulated cells from the same subset is illustrated as heatmap. (B) Bar charts with median relative MFI ± SEM, n=4 healthy donors. Click here for file
