# Supplementary material for: Phospho-specific flow cytometry identifies aberrant signaling in indolent B-cell lymphoma
Source: BMC Cancer. 2012 Oct 16;12:478. doi: 10.1186/1471-2407-12-478 (PMC3519597; doi:10.1186/1471-2407-12-478)
Supplement: Additional file 1 — Figure S1. No difference in anti-BCR induced signaling between CD20+CD5-and CD20+CD5+B cells. PBMCs (n=4) from healthy donors were stimulated with aBCR for 4, 15 or 45 minutes. Flow cytometry analysis of p-SFKs, p-SYK, p- PLCγ and p-S6 after gating on CD20+CD5+ or CD20+CD5- cells. (A) MFI in aBCR stimulated cells relative to unstimulated cells from the same subset is illustrated as heatmap. (B) Bar charts with median relative MFI ± SEM, n=4 healthy donors. [file 1471-2407-12-478-S1.pdf]

Supplemental Figure 1.

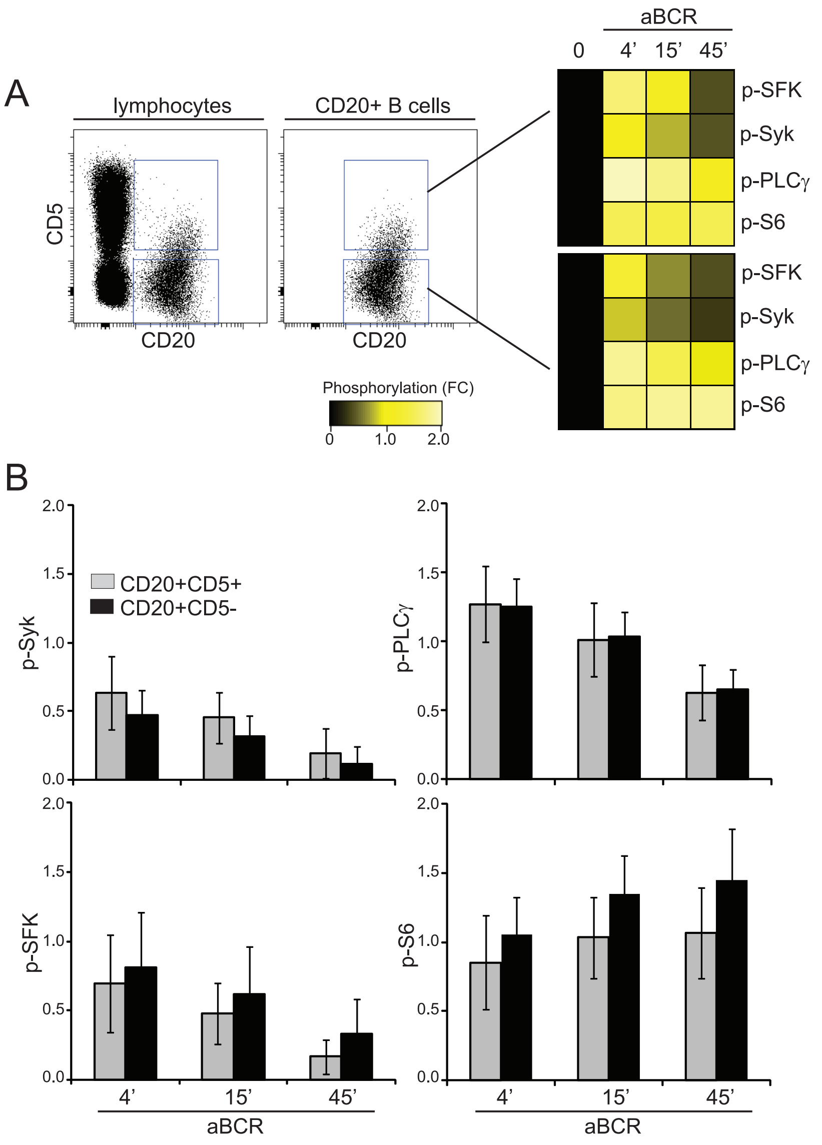

No difference in anti-BCR-induced signaling between CD20$^+$CD5$^+$ and CD20$^+$CD5$^-$ B cells. PBMCs (n=4) from healthy donors were stimulated with anti- (a)BCR for 4, 15 or 45 minutes. Flow cytometry analysis of p-SFKs, p-SYK, p- PLCγ and p-S6 after gating on CD20$^+$CD5$^+$ or CD20$^+$CD5$^-$ cells. (A) MFI in aBCR stimulated cells relative to unstimulated cells from the same subset is illustrated as heatmap. (B) Bar charts with median relative MFI ± SEM, n=4 healthy donors.
